# On the all-or-none rule of conscious perception

**DOI:** 10.3389/fnhum.2013.00387

**Published:** 2013-07-19

**Authors:** Talis Bachmann

**Affiliations:** Institute of Public Law and Institute of Psychology, University of TartuTartu, Estonia

What are the true functional underpinnings of perception, emotion, consciousness, and subconscious processes remains an unsolved question (Alivisatos et al., 2012). For example, it seems somewhat mysterious how one and the same structure of neural circuits—the human brain—sometimes allows conscious experience and sometimes does not allow it. We even do not know whether this owes to the alternating activity of different circuits, the change in the activity of the same circuits, or both.

Nobody denies accumulation of pertinent data on where and how the changes in neural activity correlate with conscious experience. For instance, specific and non-specific thalamocortical systems provide circuits for representing the contents and guaranteeing the state of consciousness (Ribary, 2005; Liu et al., 2013), processes in the higher level sensory-perceptual cortical areas tend to correlate with awareness more than the processes in the primary areas (Logothetis et al., 1996; Hesselmann et al., 2011), reentrant activity from higher to lower level nodes seems to constitute a typical feature of circuits involved in conscious perception (Lamme, 2010), etc. Yet, many principal questions have remained without answers. For example, we do not know whether conscious experience of a stimulus is a gradually emerging attribute of the activity of the corresponding neural circuits or is it an effect appearing abruptly as soon as a corresponding neural marker appears. Recently, a notable study was published trying to shed light on this unsolved problem (Sekar et al., 2013).

Sekar et al. (2013) recorded neuromagnetic responses in temporoparietal channels and found that the MEG response peaking about 240 milliseconds (ms) after stimulus reliably discriminated trials with stimulus awareness from trials lacking stimulus awareness. Importantly, this MEG-marker was expressed with an *equal* magnitude across trials where subjects reported *different subjective gradations* of stimulus experience, but was decreased when no awareness was reported. Conscious experience was interpreted as emerging according to the all or none, rule. The different subjective gradations used for sampling the trials for comparative event-related fields (ERF) analysis were “didn't see” for the unaware condition and “couldn't identify,” “unsure,” and “sure” for the three conditions with changing awareness levels. Several features of this work make it a landmark contribution to the research on neural correlates of consciousness (NCC).

A confound between physical variability of stimulation and the putative NCC-markers has been a typical obstacle for obtaining clearcut information on NCC (Bachmann, 2009). Sekar et al. (2013) kept threshold-level stimulation invariant and measured variable MEG responses as a correlate of the varying subjective responses, avoiding co-variation between the effects of varying physical stimulation and brain processes underlying subjective phenomena. Furthermore, while in the majority of earlier research on NCC objective categorical responses have been used as the dependent measure, graded subjective responses in addition to objective discrimination measures were used (Sekar et al., 2013). Obviously, temporal resolution of the brain-imaging methods suitable for studying the fast formation of conscious perception should be compatible with the corresponding fast brain processes; on the other hand, to understand the precise brain circuits involved in producing conscious perception, imaging should enable 3D analysis. In the majority of earlier research on NCC the recording methods have been either slow (fMRI, PET) or fast but controversial in terms of source analysis (EEG). Furthermore, recently it was shown that subjective experience of a stimulus can vary between “seen” and “unseen” while fMRI recording data could not differentiate between these two alternative conditions (Schoenfeld et al., 2011). Using MEG (Sekar et al., 2013) these limitations were surpassed at once. Yet, in spite of these advantages Sekar et al. paper cannot be conclusive in settling the issue about graduality vs. “quantal step like” nature of conscious perception.

(1) Because MEG recording critically depends on synchrony of (dendritic) neural activity, the accounts of consciousness mechanisms requiring no synchrony for NCC and the corresponding recording methods may suggest different solutions (He and Raichle, 2009). (2) Whether transition between states is instantaneous or gradual (“quantal” vs. “wave-like”) may depend on the time scale considered. The cellular and sub-cellular level processes responsible for awareness may lead to a gradual change of state (e.g., membrane potential) occurring at the millisecond time-scale although this may appear “instantaneous” if looked at from the timescale where the unit time is several orders higher. Although the peaks of MEG-responses indicative of stimulus awareness were statistically invariant (Sekar et al., 2013), the pre-peak ascending phase of the response was gradual covering about 50–100 ms. Even though supra-threshold stimulus-awareness may be all or none, the formation of the contents of a conscious percept may be gradual, albeit very fast. (3) Because MEG data was collected for parieto-temporal areas and analogous data about occipital sources were not reported (Sekar et al., 2013) we do not know whether earlier responses in occipital sources also would have shown analogous results. Moreover, it is uncertain whether we deal with true NCC or with markers of activity of the mechanisms controlling response choice or their top-down effects. Circuits located more dorsally are more access- and control-related compared to the temporal and occipital circuits that are more perceptual content related. A recently suggested (Aru et al., 2012; de Graaf et al., 2012) requirement to differentiate processes that are prerequisite for NCC, aftereffects of NCC, and true NCC was not followed by Sekar et al. (2013). (4) According to this study the earliest marker of the varying awareness appears at 240 ms post stimulus. However, *peak latency* need not be the only viable indicator of the process we are interested in. Instead, ERF deviation change (e.g., some critical value of derivatives of wave function) or some characteristic of ERF function's envelope spanning a time epoch inclusive of shorter delays (compared to peak delay) may show a different time-course. Moreover, when a stimulus with its energy at discrimination threshold is used, the process of percept formation typically unfolding at a faster rate may be considerably prolonged and the estimates of the fastest times with which a stimulus can reach consciousness become too much conservative. Thus, instead of the 240 ms, about 150 ms would be a correct value for the fastest delay of visual awareness. (For example, with suprathreshold, high contrast targets backward masking disappears when mask is delayed by 150 ms or more—Bachmann, 1994.) (5) Although statistical analysis on the ERF peak amplitude values of the component delayed by 240 ms showed no difference between three subjective awareness conditions, visual inspection of the corresponding magnetic flux data (Figure 4, Sekar et al., 2013) showed a systematic increase with increasing subjective estimates of the experienced contents. Thus, with more measurements statistically significant results indicative of the gradual change of ERF peak values could be obtained in principle.

Notwithstanding the debatable aspects of the Sekar et al. (2013) work, it seems a contribution providing standards for the researchers who aim at finding how neural circuits help make the processed perceptual data consciously experienced. Invariance of physical stimulation combined with variability of subjective measures, using a gradual scale of subjective measurement, and using recording and analysis methods allowing location of the circuits of interest in 3D are among the prime requirements. Of course, not all landmark publications must carry ultimate truths, but they at least show how research on NCC should be conducted in a methodologically rigorous way.
